# Atlas and Textbook of Human Anatomy

**Published:** 1907-07

**Authors:** 


### BOOKS AND AUTHORS.

**Atlas and Textbook of Human Anatomy.** Volume II. By **Professor J. Sobotta,** of Wurzburg. Edited, with additions, by **J. Playfair McMurrich,** A.M., Ph.D., Professor of Anatomy at the University of Michigan, Ann Arbor. Quarto, 194 pages, containing 214 illustrations, mostly in colors. Philadelphia and London: W. B. Saunders Company. 1906. (Cloth, $6.00; half morocco, $7.00, net.)

The simplest and best way to begin a notice of this volume is to say, as does the author, that it is a continuation of the atlas and textbook of human anatomy as begun in the first, and deals with the viscera including the heart. He further states that this arrangement seemed desirable because the heart is usually dissected in common with the other viscera.

One of the most striking features of this excellent work is the quality of the illustrations. In no previous work on human anatomy has the engraver's art been brought to such perfection, nor has printing in color been so artistically applied. In the notice given of the first volume published in the issue of the JOURNAL for February, 1907, we gave some prominence to this feature of Sobotta's work, all of which we reaffirm with emphasis, and may apply it with still more cogent force to the illustrations of the viscera. Take, for example, the figures relating to the pancreas, 392 and 393, and we fail to understand how they could depict with greater accuracy this interesting organ. We could repeat this statement with reference to all other viscera dealt with in the book, but it is quite unnecessary to do so.

The text is, also, most interesting in its dealings with the various organs of the body. One example will suffice. Sobotta divides the digestive tube into three sections,—the foregut, the midgut, and the endgut. The foregut includes the pharynx, the esophagus, and the stomach. The midgut is the small intestine, and the endgut is composed of the large intestine and rectum. The work must be found in possession of every anatomist.

